# 89723 CD105-targeted CAR T cells for the treatment of acute myeloid leukemia

**DOI:** 10.1017/cts.2021.450

**Published:** 2021-03-30

**Authors:** Konstantinos Lontos, Yiyang Wang, Andrew Frisch, Mason Colbert, Jason Lohmueller, Greg M. Delgoffe

**Affiliations:** 1 University of Pittsburgh; 2 Tsinghua University

## Abstract

ABSTRACT IMPACT: Our work might lead to a new treatment for patients with acute myeloid leukemia OBJECTIVES/GOALS: Acute myeloid leukemia (AML) is a devastating hematologic malignancy, with dismal 5-year survival. Chimeric antigen receptor (CAR) T cells have been approved for B cell malignancies but not for AML. The goal of this study is to explore the safety and efficacy of CAR T cells targeting CD105 (endoglin) to treat AML. METHODS/STUDY POPULATION: We have constructed human and murine CAR T cells targeting CD105. The CARs were created by sequencing the V(D)J regions of hybridomas and designing single chain variable fragments that target CD105 which were subsequently introduced in a CAR backbone via Gibson assembly. The CAR T cells were produced via transduction using retrovirus or lentivirus. Leukemia cell lines were assessed for CD105 expression with flow cytometry. Killing assays were performed via measurement of luminescence of target cells after co-culture with CAR T cells. Activation assays were performed with co-culture of CAR T cells and target cells and measurement of activation markers with flow cytometry. To assess in vivo efficacy and safety, murine CAR T cells were infused into C57BL/6J mice carrying B16 melanoma after lymphodepletion. RESULTS/ANTICIPATED RESULTS: All human leukemia cell lines assessed (Nalm6, MOLM-14, MV4-11, Kasumi-1, THP-1) expressed some degree of endoglin apart from the T cell leukemia Jurkat. Human CD105 CAR T cells were activated by co-culture with leukemia cell lines and effectively killed leukemia cells in vitro in a CD105-specific manner. Murine CAR T cells killed efficiently both murine solid tumors (B16 melanoma) and murine leukemias (C1498) in vitro. Murine CAR T cells did not exhibit any toxicity when infused after low-dose lymphodepletion (cyclophosphamide 100mg/kg) but caused significant morbidity after higher doses (cyclophosphamide 200mg/kg). Murine CAR T cells delayed the growth of B16 melanoma in immunocompetent mice. DISCUSSION/SIGNIFICANCE OF FINDINGS: We have constructed human and murine CD105 CAR T cells with excellent activity in vitro. The activity of human CD105 CAR T cells in xenografts and the biologic relevance of the toxicity of murine CD105 CAR T cells in humans needs to be further investigated. CD105 CAR T cells might prove an important therapeutic option for patients with AML.

